# Current advances in cancer immunohistochemistry: a new perspective for the Ki-67 biomarker

**DOI:** 10.3332/ecancer.2025.1863

**Published:** 2025-03-05

**Authors:** Talita Alves do Nascimento Santos, Anna Karoline Fausto da Silva, Karin Soares Gonçalves Cunha, Camila Braz Pereira da Costa, Aldo Rodrigues da Silva, Helena Carla Castro, Nathália Silva Carlos Oliveira

**Affiliations:** 1 Programa de Pós-Graduação em Patologia, Faculdade de Medicina, Hospital Universitário Antônio Pedro, Universidade Federal Fluminense, Niteroi, RJ 24033-900, Brazil; 2 Serviço de Anatomia Patológica, Hospital Universitário Antônio Pedro, Universidade Federal Fluminense, Niteroi, RJ 24033-900, Brazil.; 3 Departamento de Patologia, Faculdade de Medicina, Hospital Universitário Antônio Pedro, Universidade Federal Fluminense, Niteroi, RJ 24033-900, Brazil; 4 Diretoria Industrial, Instituto Vital Brazil, Niteroi, RJ 24230-410, Brazil; 5 LABiEMOL - PPBI - Departamento de Biologia Molecular e Celular, Instituto de Biologia, Universidade Federal Fluminense, Niteroi, RJ 24210-201, Brazil

**Keywords:** Ki-67, immunohistochemistry, cell proliferation, biomarker, immunostaining, nuclear protein, personalised medicine, neoplasia, histopathological diagnostic

## Abstract

Ki-67 is a cell proliferation biomarker used to evaluate the proliferative activity of neoplasia cells. However, considering its functions on the cell cycle, the standard method seems to be an underused way of evaluating expression, since so far, its analytical validity of Ki-67 remains questionable for its use in personalised therapy. Improvements in the assessment of Ki-67 expression continue to be explored, and recently, a new approach that considers the heterogeneity or variability in staining intensity has emerged as a more improved way than the traditional method. In this review, we bring together what is available in the literature on the biological properties of the protein and highlight how this potential association is promising in the field of personalised medicine.

## Trial registration

Since this is a narrative review with no meta-analysis, it does not involve the conduction of new clinical trials, patient recruitment or primary data collection. Therefore, it falls outside the scope of studies requiring trial registration in databases such ClinicalTrials.gov or WHOICTRP.

## Introduction

Cell proliferation is a fundamental process critical for tissue homeostasis, driven by tightly regulated and coordinated events that ensure accurate segregation and transmission of genetic material to daughter cells. However, disruptions in the regulatory mechanisms of the cell cycle can lead to uncontrolled proliferation, resulting in pathological conditions such as neoplasms [1].

Malignant neoplasms are a significant global health challenge, with the World Health Organization reporting approximately 10 million deaths annually, making cancer the second leading cause of mortality worldwide [2]. Despite advancements in prevention, treatment strategies and understanding tumour biology, early diagnosis remains the most effective means to improve clinical outcomes and, unfortunately, many cases are diagnosed at advanced stages [3].

In this context, precision medicine [4] has emerged as a promising approach for personalizing therapies and improving patient survival [5]. Genes associated with cell proliferation are notably upregulated during the cell cycle, and the detection of their protein products has become an invaluable diagnostic and prognostic tool. Among these, the nuclear protein Ki-67 stands out. Expressed during all active phases of the cell cycle – except in resting cells and early G1 – it is widely used in anatomopathological analyses to assess the growth fraction of cell populations [6].

The utility of Ki-67 extends beyond its role in monitoring cell cycle activity. It is routinely detected through standard immunodetection techniques such as immunohistochemistry and immunocytochemistry [7]. The labelling index (LI), calculated as the percentage of Ki-67-positive cells relative to the total cell count in a sample, provides insights into tumour growth dynamics. Together with the mitotic index (the count of cells in mitosis), the LI offers estimates of tumour aggressiveness and malignancy, contributing to the classification of tumours [8]. Furthermore, Ki-67 expression can serve as a prognostic marker, indicating tumour progression or outcomes and a predictive marker, forecasting recurrence, progression or mortality risk [9].

The prognostic and predictive significance of Ki-67 has been established for several malignancies [10, 11], including non-Hodgkin lymphomas [12], renal carcinoma [13], soft tissue sarcomas [14], prostate carcinoma [15], breast cancer [9] and neuroendocrine tumours (NETs) [16]. Intriguingly, variations in both the intensity and nuclear distribution of Ki-67 expression have been observed during histopathological evaluations, though these heterogeneities are often overlooked in routine diagnostics [17].

Emerging evidence highlights that the functions of Ki-67 and nuclear localization vary depending on the cell cycle phase, offering a nuanced view of cell cycle dynamics [18]. This phase-specific distribution underscores its potential as a biomarker not just for proliferation but also for cell cycle status. Harnessing these characteristics could pave the way for personalised antitumour therapies, enabling phase-specific classifications of proliferative cells. This approach could refine the use of Ki-67 in modulating treatment strategies, enhancing its relevance in precision oncology.

### KI-67: molecular and genetics characteristics

The Ki-67 protein is encoded by the MKI67 gene, located on chromosome 10q25.qter in humans. This gene spans approximately 29,965 base pairs and consists of 15 exons and 14 introns [19]. Its transcription produces multiple isoforms through alternative splicing, including Ki-67 α, β, δ, ε and γ [20]. Among these, the α and β isoforms are detected in cancer cells, with the β isoform being exclusively found in normal cells, linked to cell cycle progression [21]. The functions of other isoforms remain underexplored.

Structurally, Ki-67 includes distinct functional domains.

- N-terminal domain: It features a forkhead-associated domain involved in chromatin regulation and early ribosomal RNA synthesis, along with a binding site for protein phosphatase 1, which modulates chromatin dynamics.
- Central region: It contains 16 disordered repeats (‘Ki-repeats’) that may contribute to phase separation.
- C-terminal domain: It contains a leucine-rich region essential for chromosomal association during mitosis [13].

The MKI67 promoter includes regulatory elements such as cell cycle-dependent elements (CDEs) and cell cycle homology regions (CHRs), typical of late cell cycle genes [22]. Although mRNA levels of MKI67 are similar across cell lines, transcription is phase-regulated [23]. The DREAM complex, which interacts with the CDE and CHR regions, plays a pivotal role in gene activation and repression. Specifically, the following conditions hold.

1. In G0 and early G1, the RBL1-DREAM-MuvB repressor complex binds the promoter and inhibits transcription [24].
2. After mitogenic signals, cyclin D-CDK4/6 phosphorylates RBL1, dissociating the repressor complex, but transcription remains inactive [24, 25].
3. At the G1/S transition, the MuvB complex recruits the BMyB protein, activating transcription [26].
4. In G2, further recruitment of FoxM1 to the MuvB-BMyB complex enhances transcription until mitosis [26].

The dynamics of MKI67 transcription differ between normal and cancerous cells [27]. For instance, MKI67 expression remains constant in cancer cells without evident mutations, but variations in transcription patterns during the S phase have been observed across different cell lines [27–31]. These discrepancies may arise from differences in experimental protocols or the pathways involved in G0-G1 and Mitosis-G1 transitions, warranting further investigation.

Protein kinetics also reveal that Ki-67 has a short half-life (~1 hour) [32], with degradation mediated by the anaphase-promoting complex (APC/C-Cdh1) in G0, early G1 and late mitosis [23]. Recent findings suggest that Ki-67 ubiquitination in post-mitotic neurons involves the APC7 subunit, potentially linking Ki-67 to neurological conditions [33]. Enhanced understanding of Ki-67 degradation and expression variability during mitosis and G1 could provide insights into its role in cell cycle regulation [33].

In conclusion, while the transcriptional regulation and structural domains of Ki-67 are increasingly understood, further studies are needed to elucidate its isoform-specific roles, variability in expression patterns and degradation mechanisms in normal and pathological conditions.

### KI-67 function and distribution during the cell cycle

Ki-67 demonstrates dynamic localization and functional roles throughout the cell cycle, regulated by post-translational modifications, particularly phosphorylation. In the early interphase, Ki-67 is dephosphorylated, allowing DNA binding at satellite regions. As the nuclear bodies coalesce and nucleoli form, Ki-67 interacts with nucleolar protein NIFKh, relocating to the nucleolar cortex and forming a reticulated structure. During the S and G2 phases, Ki-67 mRNA transcription increases, with the protein accumulating in the nucleoli and nucleoplasm, particularly as cells approach mitosis [34].

At mitosis onset, cyclin B-Cdk1 phosphorylates Ki-67 at specific sites, altering its charge and dissociating it from DNA, facilitating its migration to the chromosome periphery to form the perichromosomal layer. This dynamic process, influenced by interactions with kinesin-like motor proteins, is reversed at mitosis completion, restoring nonphosphorylated state of Ki-67 and its DNA-binding capacity [34–37].

The localization of Ki-67 shifts during the cell cycle has been extensively documented using *in vitro* models [28, 30, 32, 34, 38–40]. During interphase, it organises heterochromatin [41], guides nucleolar organizing regions (NORs) [40] and participates in ribosome biogenesis [42, 43]. In mitosis, it aids in perichromosomal layer formation, chromosomal mobility and mitotic chromosome clustering [18, 35, 43, 44]. Morphological and functional variations are evident, such as Ki-67 granules in G1 and nucleoplasm-wide distribution in the late S phase [40, 41].

**Figure 1.**
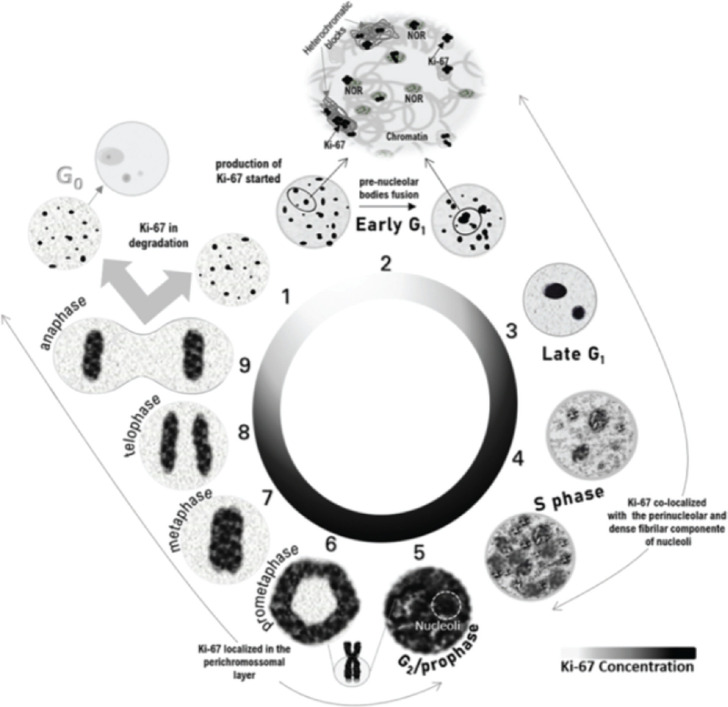
Schematic representation of Ki-67 localization throughout the cell cycle based on previously published experiments. During the early G1 phase, Ki-67 remains from the previous cell cycle located in the heterochromatic blocks and co-localises with the NORs. After the fusion of NORs, Ki-67 can be identified in the nucleoli. At the end of the G1 phase, the small nucleoli gather into one or two larger nucleoli and Ki-67 is localised in the nucleoli as discrete points in the nucleoplasm. At the beginning of the S phase, Ki-67 continues to be in the nucleoli and appears as coarser granules throughout the nucleoplasm, filling a large part of the nucleoplasm at the end of the S phase. During the G2 phase and until prophase, the nucleoli are barely evident due to the almost complete filling of the nucleoplasm by Ki-67, coinciding with the maximum expression of MKI67. From prometaphase to anaphase, Ki-67 is relocated to the structure of mitotic chromosomes. Cells that enter rest after mitosis have degraded Ki-67.

Notably, discrepancies in S and G2 phase patterns among cell lines suggest methodological influences or intrinsic variability in Ki-67 expression [17, 28]. Mitosis is the most consistently described phase, where Ki-67 localises to the chromosomal periphery [18, 43], facilitating chromosome segregation [39] and nucleolar reassembly [45]. The protein either returns to heterochromatin in cycling cells or degrades in quiescent cells [23]. Based on published data, distinct Ki-67 morphological patterns correlate with specific cell cycle phases, Figure 1, reinforcing its utility as a biomarker for cellular dynamics and therapeutic targeting.

### KI-67: histopathological application and new analysis methods

The Ki-67 labelling index is a widely used method in histopathology to assess tumour proliferative activity and determine its aggressiveness. This method involves counting the number of Ki-67-positive cells relative to the total number of cells within a predefined area. The percentage of positive cells is calculated by multiplying the result by 100 and Ki-67 positive is often classified using cutoff points, which stratify proliferative activity as low, medium or high [46, 47]. This allows for the determination of malignancy status and, in some cases, provides prognostic or predictive value [48].

Although workgroups have developed guidelines for Ki-67 usage and specified cutoff points based on tumour type, these guidelines require regular review and updates [30, 47–49]. Traditionally, Ki-67 quantification relies on a binary system (positive or negative), but several alternative methods exist, including visual estimation using a microscope, manual counting of digital images and automated analysis using machine learning algorithms. However, a consensus on the most suitable method has yet to be established [49].

One challenge in validating Ki-67 analytically is the heterogeneity of nuclear immunostaining, which varies with its localization during the cell cycle. Traditional binary analyses overlook these variations, despite evidence from several studies showing that they provide valuable insights into the function of Ki-67 throughout the cell cycle [49, 50]. Recognizing this, recent research has proposed innovative approaches to evaluate Ki-67 expression, accounting for its graduated nature.

**Figure 2.**
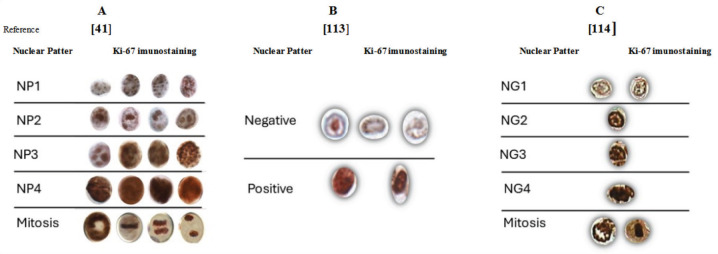
Comparison of the representation of Ki-67 patterns immunostaining. (a): Nuclear patterns categorised according to the cell cycle phases. (b): Nucleus categorised in negative or positive according to the immunostaining intensity. (c): Nucleus categorised by the nuclear gradient according to the cell cycle phases, chromatin texture and cell cycle phase.

A pioneering study introduced a theoretical model linking nuclear patterns to cell cycle phases, classifying Ki-67 staining into five distinct patterns (NP1–NP5). This approach demonstrated that specific nuclear patterns correlate with cell cycle phases, providing a nuanced understanding of proliferative activity Table 1, Figure 2A. For example, NP1 was predominantly observed in tissues without lesions or with hyperplasia, indicative of cells in the G1 phase [17].

In contrast, carcinomas displayed higher proportions of cells with NP4 staining, representing the G2/M phase. The method showed high interobserver reliability and moderate diagnostic agreement. In addition, haematoxylin–eosin staining improved mitotic phase identification, except for prophase, which overlapped with G2 phase cells [17].

Another study focused on the intensity of Ki-67 staining in NET samples, disregarding localization. By considering only intensely stained nuclei (associated with the G2/M phase), this approach reduced false negatives seen in traditional methods. It resulted in up to a 30% reduction in high-grade tumour classifications and demonstrated potential predictive value for survival rates in NET patients [52] Table 1, Figure 2B.

A third study integrated nuclear morphometry, chromatin texture, staining intensity and localization to create a nuclear gradient classification system. This semiautomated method enabled the correlation of Ki-67 nuclear gradients with tumour activity and prognosis in pulmonary typical carcinoma Table 1, Figure 2C. High and low gradients were visualised using heat maps, underscoring the utility of this technique in diagnosis. While advanced techniques, such as flow cytometry, multiplex immunohistochemistry and FUCCI fluorescence, provide valuable cell cycle data, their limitations include high costs, time consumption and reliance on specialised equipment and expertise. Moreover, these techniques often fail to capture intratumoural heterogeneity due to extensive cellular manipulation [53].

An alternative method involves analysing DNA content with DAPI, which considers differences in DNA texture, content and shape to identify cell cycle phases. However, challenges remain in accurately distinguishing transitions between G1/S, S/G2 and G2/M phases. To address these limitations, further research into the relationship between Ki-67 morphological patterns and cell cycle phases in formalin-fixed paraffin-embedded samples holds promise. This approach leverages a widely used marker in routine histopathology, offering the potential for improved classification of cell cycle phases using a single, accessible marker.

Therefore, to enhance the practical applications of Ki-67 in histopathological analyses, beyond its conventional usage, new methodologies and approaches have emerged to refine its diagnostic and prognostic capabilities, and some of these uses and advantages are summarised in Table 2.

By integrating these advancements into routine practice, Ki-67 assessment not only remains a cornerstone of tumour biology but also evolves to meet the demands of precision medicine, ultimately improving diagnostic accuracy and patient outcomes. Therefore, we are faced with the possibility of classifying the phases of the cell cycle using a single marker, already known and widely used in routine histopathology (Table 2).

## Discussion

In this review, we described studies that demonstrated new methods for semiquantitative evaluation of the immunohistochemical expression of Ki-67. It discusses innovative methods that account for the variability in Ki-67 expression and localization throughout the cell cycle, enabling the categorization of morphological patterns in formalin-fixed, paraffin-embedded tissues. The proposed classification systems incorporate morphological and morphometric characteristics, along with the heterogeneity of Ki-67 immunostaining. They can be broadly divided into two categories: one based on differences in protein localization and intensity during cell cycle phases and the other solely focused on variations in staining intensity [17, 52, 53].

The first approach highlights the biomarker potential for mapping cell cycle dynamics, while the second aims to mitigate the impact of immunostaining heterogeneity on tumour stratification. Both approaches demonstrated positive outcomes when compared to the gold standard, including improvements in histological classification, prognostic definition and clinical outcome prediction.

Mapping cell cycle dynamics is crucial, particularly in cancer – a complex and heterogeneous disease influenced by metabolic, genetic, epigenetic, molecular and microenvironmental factors. Tumour heterogeneity complicates classification, prognosis and therapeutic decisions, contributing to recurrence and metastasis [50]. As cancer is fundamentally a disorder of cell proliferation, cell cycle-specific chemotherapies represent a viable treatment strategy [51]. However, effective application requires accurate mapping of cell cycle dynamics [54, 55]. While immunohistochemistry is useful for cell cycle characterization, relying on specific markers for each phase is labour-intensive and costly [54]. Similarly, advanced techniques, such as fluorescence *in situ* hybridization, next-generation sequencing and single-cell sequencing, though valuable, are prohibitively expensive for routine diagnosis [56–58].

The proposed new methods simplify this process by leveraging a single, well-established biomarker, Ki-67, already widely used in routine histopathology. These methods offer advantages in time and cost, essential factors in reducing cancer-related morbidity and mortality. Improvements in Ki-67 evaluation have enhanced its diagnostic, prognostic and predictive value, presenting new opportunities for personalised therapy. Future studies should prioritise validating these methods in diverse tissue types, both normal and neoplastic, using multiplex immunohistochemistry to explore their full potential.

One challenge in Ki-67 quantification arises from overestimating positive nuclei when following WHO guidelines, which count any Ki-67-positive cell. This can lead to misclassification, as resting or slow-growing cells with residual Ki-67 may be erroneously included as actively proliferating [sobecki]. To address this, some studies propose a classification based on staining intensity, aiming to reduce false positives. While this approach has shown promise, especially in NET, it requires caution. Protein accumulation over multiple cell cycles poses challenges in setting accurate thresholds, yet this method may help identify slow-growing cells, which are associated with chemotherapy resistance, recurrence and metastasis.

Finally, understanding Ki-67 dynamics could bridge gaps in cancer characterization. By refining its application in personalised therapy, Ki-67 has the potential to become a robust clinicopathological biomarker. Future studies should focus on validating these innovative methods in formalin-fixed, paraffin-embedded tissues across various proliferative disorders. With its widespread use, low cost and ease of implementation, Ki-67 remains a promising tool for advancing histopathological and therapeutic precision.

## Conclusion

In conclusion, this review highlights new methods proposed for analysing Ki-67 expression in routine histopathology. By considering the diverse nuclear patterns and distribution of Ki-67 across cell cycle phases, these methods demonstrated advantages over the traditional gold standard approach. Notably, they raise the possibility of mapping cell cycle dynamics by correlating Ki-67 patterns with specific phases. However, as these methods rely on empirical models, further research is required to explore their full potential.

Given that the theoretical foundation was derived from studies on cell lines and various techniques, it is recommended to validate these models in formalin-fixed, paraffin-embedded tissues. Applying multiplex immunohistochemistry to samples representing different proliferative states (normal,

hyperplastic, dysplastic, metaplastic and neoplastic) could provide critical insights. Such validation would extend the applicability of these methods, potentially revolutionizing routine histopathology.

The widespread use of Ki-67 in clinical settings is attributed to its low cost, ease of use and minimal requirements for specialised equipment or advanced training. These characteristics align well with its integration into standard histopathological practices. Consequently, Ki-67 holds significant promise as a clinicopathological biomarker with potential applications in personalised therapy. Validating innovative methodologies to enhance its utility could make Ki-67 a cornerstone in tailoring cancer diagnostics and treatment strategies.

## List of abbreviations

**APC7**, A**naphase-promoting complex subunit 7**; **APC/C-Cdh1**, A**naphase-promoting complex Cdh1**; **CDE**, C**ell cycle dependent elements**;** CHR**, C**ell cycle genes homology regions**; FUCCI, Fluorescent cell cycle indicator; **Hklp2**, K**inesin-like motor protein**; **LI**, L**abe**ll**ing index**; **NET**,** Neuroendocrine tumours**; NG, Nuclear gradient; **NIFKh**,** Human nucleolar protein**; **NORs**,** Nucleolar organi**s**er regions**.

## Conflicts of interest

The authors declare no conflicts of interest.

## Funding

The authors have not received any kind of funding for the research and writing of this paper.

## Consent for publication

All the authors of the article agreed to be published in the journal.

## Author contributions

All authors contributed to the conception of the study. TANSD wrote this paper, and AKFS, NSCO, KSGC, CBPCS, HCC and ARS reviewed the paper. All authors had access to the study data and reviewed and approved the final manuscript.

## Figures and Tables

**Table 1.** Comparison of classifications for immunohistochemistry cited by the authors.

Reference	Nuclear patterns	Cell cycle phase relative	Nuclei size	Nucleoplasm	Nucleoli	Ki-67 immunostained
**[17]**	PN1	G1 early	Normal	Clear	Absent	Colocalised with pre-nucleolar corpuscles visible as discrete granules.
	PN2	G1 late	Normal	Slightly immunostained	One or two central nodes	Colocalised with the nucleoli (in the form of nodules), distributed as granules throughout the nucleoplasm and surrounding the nuclear periphery
	PN3	S	Increased	Fully immunostained	One or two central nodes	Present in the nucleoli in the form of nodules and distributed throughout the nucleoplasm in the form of coarse granules, filling it completely or partially
	PN4	G2	Increased	Fully immunostained	Not visible	Homogeneous as a single spot throughout the nucleoplasm
	Mitosis	Prophase	Increased	Fully immunostained	Not visible	Homogeneous as a single spot throughout the nucleoplasm
		Prometaphase	circular with a void in the centre	Discreetly immunostained	Absent	Circular with a void in the centre
						Exclusively on the chromosome
		Metaphase	Not delimited by membrane			
		Anaphase				
		Telophase				
**[52]**	Negative	G0, G1, S	Normal	Clear	Absent	Light brown
	Positive	G2/M	Normal	Slightly immunostaining	One or two central nodes	Dark brown or black
**[53]**	NG1	G0	Normal	absent	Fine or coarse granules and nuclear membrane	Fine or coarse granules
	NG2	G1	Normal		Perinucleolar area and nuclear membrane	One or two nodes, perinucleolar area and nuclear membrane positivity
	NG3/NG4	S/G2	Increased volume and anisocariosis	One or two central nodes	Perinucleolar Heterochromatin and nucleolus	Intense and homogeneous in nucleoplasm and nucleoli
	Mitosis	Prophase	Mitotic nuclei	Not visible	Perinucleolar regions, nucleolus and nucleoplasm	Empty central area and the presence of random peripheral Ki-67 immunostained granules/nodules.
		Prometaphase	Mitotic nuclei	Not visible	Perichromossomal	Exclusively on the chromosome
		Metaphase		Absent	Perichromossomal	
		Anaphase		Absent	Exclusively on the chromosome	
		Telophase		Absent	Exclusively on the chromosome	

**Table 2.** List of uses and advantages of practical applications of Ki-67 in histopathological analyses.

Use	Advantages
Enhanced tumour classification	Recent studies suggest the potential for using Ki-67 nuclear localization and staining intensity as a phase-specific marker of the cell cycle. This granular approach allows for the identification of cell cycle phases within paraffin-embedded tissue, offering insights into tumour growth dynamics and phenotypic heterogeneity. Such advancements may improve the classification of tumours, enabling more precise stratification of malignancy grades.
Predictive value in therapeutic response	By correlating Ki-67 nuclear patterns with specific phases of the cell cycle, clinicians can gain insights into tumour responsiveness to therapies targeting proliferative cells. For instance, higher proportions of cells in the G2/M phase, marked by specific Ki-67 patterns, may indicate a more aggressive tumour phenotype and guide therapeutic decisions.
Automated and semiautomated quantification techniques	Emerging technologies, such as machine learning algorithms for automated counting and nuclear gradient mapping, improve the reproducibility and accuracy of Ki-67 quantification. These methods reduce interobserver variability and offer robust tools for routine diagnostics, particularly in high-throughput laboratory settings.
Integration with advanced imaging and molecular profiling	Techniques, such as multiplex immunohistochemistry and fluorescence-based cell cycle indicators, though cost-intensive, enhance the utility of Ki-67 by providing complementary data on cell cycle dynamics. Combining Ki-67 analysis with advanced imaging systems could enable spatial mapping of proliferative activity within tumour microenvironments, aiding in the characterization of intratumoural heterogeneity
Clinical relevance in specific cancers	Refinements in Ki-67 evaluation methods have shown significant promise in cancers such as breast cancer, NETs and pulmonary carcinomas. Tailored cutoff points, considering staining intensity and localization, have demonstrated improved prognostic accuracy and therapeutic stratification, especially in challenging cases like high-grade NETs.
Educational and training tools	Standardizing the interpretation of Ki-67 patterns and their correlation with clinical outcomes could form the basis for training pathologists. Digital tools incorporating annotated examples of Ki-67 nuclear patterns can enhance understanding and application, ensuring consistency in clinical practice.
Research and development of alternative biomarkers	The ongoing refinement of Ki-67 as a proliferative marker also supports its integration into multimarker panels. These panels, combining Ki-67 with markers for apoptosis, angiogenesis or immune response, could provide a more comprehensive view of tumour biology and patient prognosis
